# Atomic/molecular layer deposition of p-type conducting copper–sulfur–organic coordination polymer thin films for thermoelectric applications

**DOI:** 10.1039/d6ta01174h

**Published:** 2026-03-11

**Authors:** Mari Heikkinen, Malar Auxilia Francis, Kristoffer Meinander, Girish C. Tewari, Mikko Nisula, Maarit Karppinen

**Affiliations:** a Department of Chemistry and Materials Science, Aalto University FI-00076 Espoo Finland maarit.karppinen@aalto.fi; b Department of Bioproducts and Biosystems, Aalto University FI-00076 Espoo Finland

## Abstract

Here we report a three-precursor atomic/molecular layer deposition (ALD/MLD) process for the fabrication of copper-based coordination polymer thin films for thermoelectric applications. This process mimics the wet chemical synthesis of poly[metal-ethenetetrathiolate] (poly[M-ETT]) polymers based on a trans-metalation reaction. In our ALD/MLD process the Cu-for-Li trans-metalation is realized upon the pulsing of the three precursors, 1,3,4,6-tetrathiapentalene-2,5-dione (TPD), lithium hexamethyldisilazide (Li-HMDS) and copper(ii) acetylacetonate (Cu(acac)_2_), in a cyclic manner. The process yields p-type electrically conducting poly[Cu-ETT] thin films with an appreciably high growth-per-cycle (GPC) of ∼11 Å per cycle at a deposition temperature of 220 °C. The targeted chemical composition was confirmed with XPS measurements, which verified the Cu : S ratio at 0.246 (*i.e.* very close to the ideal 0.25 value). From electrical transport measurements, the room-temperature resistivity and Seebeck coefficient values were determined to be 0.17 Ω m^−1^ and 88 µV K^−1^, respectively.

## Abstract 2

A three-precursor ALD/MLD process based on Cu-for-Li trans-metalation reaction allows the fabrication of p-type thermoelectric poly[Cu-ETT] coordination polymer thin films with appreciably high Seebeck coefficient.

## Graphical abstract

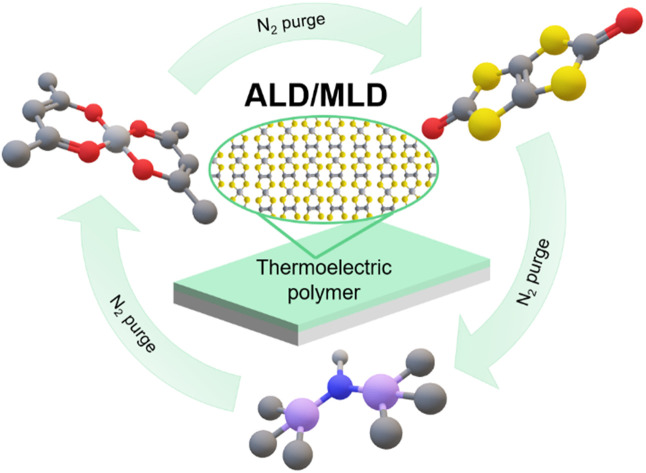

## Introduction

Mechanically flexible thermoelectric (TE) materials based on non-toxic, light-weight and cost-effective elements are in demand, *e.g.*, for self-powered wearable electronics.^1–8^ Organic TE materials such as amorphous polymers tick these boxes, but the drawback is their lack of sufficient intrinsic electrical conductivity leading to – in comparison to their crystalline inorganic counterparts – inferior TE performance, evaluated on the basis of the so-called figure-of-merit (*ZT*). Enhancements in this dimensionless *ZT* (= *S*^2^*σT*/*κ*) value are achieved by either increasing the power factor (PF = *S*^2^*σ*), which is a product of the Seebeck coefficient (*S*) and electrical conductivity (*σ*), or by lowering the thermal conductivity (*κ*). Moreover, for practical TE generators, both p- and n-type conductors are needed; these materials should be mutually compatible regarding their thermoelectric, thermal and mechanical characteristics.

Organic materials show – rather characteristically – appreciably low thermal conductivity values.^9,10^ Therefore, the main challenge is to increase the intrinsically low electrical conductivity values of organics. A promising strategy to enhance the electrical conductivity is to fuse these materials with transition metals.^11^ In particular, metal–organic coordination polymers could provide a solution that combines the mechanical flexibility, non-toxicity and intrinsically low thermal conductivity of organics with the higher electrical conductivity achieved by the incorporation of transition metal species into the polymer matrix.^12–14^

Metal-ethenetetrathiolate (poly[M-ETT]) coordination polymers are cost-effective and have shown promising TE properties in bulk material/pressed pellet form. Moreover, both p- and n-type poly[M-ETT] materials are known depending on the 3d-transition metal (M) used.^14–26^ The first reports on the poly[M-ETT] coordination polymers (M = Cu, Ni, Pt, Mn, Co, Fe, Au) are from the 1980s,^27–29^ see Table S1 in the SI. More recently, another related coordination polymer family, poly[metal-tetrathiooxalate] (poly[M-TTO]), has been highlighted for their even more promising TE characteristics; the two families differ regarding the nominal charge and nominal carbon–carbon bond order of the organic backbone, such that TTO^2−^ contains C–C and ETT^4−^ C=C bonded carbon.^15,16,30^ This organic backbone consists of electron-rich sulfur atoms along with alternating single and double bonds in the pentalene ring that provide a high degree of conjugation, believed to be beneficial for the electrical conductivity.

To fully comply with the wearable TE applications, the device dimensions should be tunable and adjustable to irregular/curved heat sources such as human skin. These requirements underline the importance of developing optimal thin-film fabrication techniques for the TE coordination polymer materials. In this aspect, the poor solubility of the poly[M-ETT] and poly[M-TTO] materials is a clear drawback, as it has made the solution-processing of these coordination polymers into flexible thin films challenging.^20,31–34^

Here we present the atomic/molecular layer deposition (ALD/MLD) technique – a state-of-the-art gas-phase technique for inorganic–organic thin films^35^ – as a viable alternative for the fabrication of poly[M-ETT] thin films for potential wearable TE applications. This technique relies on sequential pulsing of gaseous precursors and self-saturating gas-to-surface reactions. The parent ALD (atomic layer deposition) technique has already been employed as a feasible fabrication method for inorganic thermoelectrics,^36–39^ and the combined ALD/MLD technique could extend the apparent benefits of the ALD technique (such as accurate film thickness control, large-area homogeneity, excellent conformality, and precise interface control)^35,40,41^ to the metal–organic coordination polymer thermoelectrics. On top of these benefits, the introduction of organic components into the ALD-grown inorganic films through MLD cycles would significantly enhance the mechanical properties.^42,43^ In our previous studies we have already demonstrated the usability of the ALD/MLD technique for the fabrication of TE ZnO:organic superlattice thin films (of n-type conductivity).^11,41,44,45^ Namely, through smart design of the organic component itself and the frequency pattern through which it is introduced within the ALD-grown ZnO layers, the thermal conductivity of ZnO films could be suppressed by a factor of 50 without compromising the electrical transport properties.^44^ Another unique benefit of the ALD/MLD-grown ZnO:organic thin films is the fact that, through ALD/MLD, the TE film is deposited in a conformal manner on textile fibers so that the entire textile piece may become an active part of the device; this is highly promising considering the potential wearable TE devices.^46,47^

**Fig. 1:**
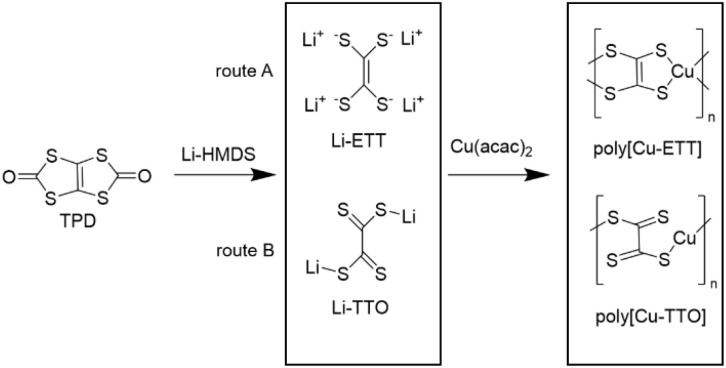
A scheme showing the possible reaction pathways for the ALD/MLD grown copper–sulfur–organic coordination polymer thin films studied in this work.

In the present study, we have developed a novel three-precursor route for the fabrication of conducting copper-ethenetetrathiolate thin films *via* ALD/MLD, using 1,3,4,6-tetrathiapentalene-2,5-dione (TPD) as the organic precursor. In our process, lithium hexamethyldisilazide (LiN(SiMe_3_)_2_; Li-HMDS) was used as a ring-opening reactant for the TPD molecule to create intermediate Li-ETT or Li-TTO species; copper(ii) acetylacetonate (Cu(acac)_2_) was then used as the copper precursor for the Cu-for-Li metal-ion exchange, or so-called trans-metalation reaction. The targeted overall reaction scheme is shown in Fig. 1. Depending on the intermediate species, the final coordination polymer product could be either poly[Cu-ETT] or poly[Cu-TTO]; in our case we believe the reaction proceeds *via* route A so that the end product is poly[Cu-ETT].^14,15^ Most excitingly, we will demonstrate that the thin films thus deposited at 220 °C are p-type electrical conductors with an appreciably high Seebeck coefficient.

## Experimental

All the present coordination thin films were grown using the following commercial precursors: 1,3,4,6-tetrathiapentalene-2,5-dione (TPD; BLDPharm, 97%), lithium hexamethyldisilazide LiN(SiMe_3_)_2_ (Li-HMDS; Sigma Aldrich, 97%), and copper(ii) acetylacetonate (Cu(acac)_2_; Thermo Scientific, 98%). The depositions were carried out in a flow-type hot-wall ALD reactor (ASM Microchemistry F-120) with a base pressure of ∼3 mbar. The precursors were heated in open boats inside the reactor for sublimation at temperatures of 95, 65 and 130 °C for TPD, Li-HMDS and Cu(acac)_2_, respectively. The deposition (substrate heating) temperature and the precursor pulse times were investigated and optimized in the study. Nitrogen (>99.999%, Schmidlin UHPN 3000 N_2_ generator) was used both as a carrier and purging gas with a flow rate of 300 sccm. The films were deposited on 2 × 2 cm^2^ Si (100) (Okmetic Ltd) and borosilicate substrates.

Process optimization by varying the pulse time of one precursor while keeping the other two fixed was carried out at a deposition temperature of 220 °C. The film thicknesses were determined with scanning electron microscopy (SEM; JEOL) by cutting the silicon substrate on which the film had been deposited into half and observing the cross section. The amorphous structure was verified for all the films using grazing-incidence X-ray diffraction (GIXRD; Rigaku SmartLab diffractometer; parallel beam; Cu Kα_1+2_) measurements; to maximize the intensity, no monochromator was used. The GIXRD patterns were collected in the 2*θ* range of 10–80° using 5.0° incident and Soller slits (limiting axial divergence).

The chemical composition and the bonding structure of the films were investigated using Fourier-transform infrared spectroscopy (FTIR) and X-ray photoelectron spectroscopy (XPS). The FTIR measurements were performed in transmission mode in the wavelength range of 400–4000 cm^−1^ with 4 cm^−1^ resolution. The spectrum obtained for a blank Si substrate was subtracted from the measured sample spectra. The XPS measurements were performed with a Kratos AXIS Ultra DLD X-ray photoelectron spectrometer using a monochromated Al Kα X-ray source (1486.7 eV) run at 100 W. Argon sputtering was not used to guarantee realistic observation of the copper oxidation state. A pass energy of 80 eV and a step size of 1.0 eV were used for the survey spectra, while a pass energy of 20 eV and a step size of 0.1 eV were used for the core-level spectra. Both survey and core-level spectra were collected from three different spots on the thin-film sample surface to check the homogeneity and surface charge effects. No charging effects were seen on the sample. All spectra were charge-corrected relative to the position of C–C bonding of carbon at 284.8 eV.

Electrical transport measurements (resistivity and Seebeck coefficient) were performed using a physical property measurement system (PPMS; Quantum Design; 9 T magnet) equipped with a thermal transport option (TTO). Four probes were attached to a rectangular thin film sample on a glass substrate; a heater was attached to one end probe and two thermometers to the middle probes from which Seebeck voltage and temperature difference were measured simultaneously.

## Results and discussion

Among the three precursors, *i.e.* Cu(acac)_2_, Li-HMDS and TPD, used in this study for the growth of the Cu-based coordination polymer thin films, the two metal precursors had been utilized earlier in different ALD and ALD/MLD processes, but the organic precursor TPD had not been used in ALD/MLD before.^35,48^ The carbonyl groups in TPD were expected to be of moderate reactivity towards conventional ALD metal precursors;^49^ hence, we first screened the reactivity of TPD in combination with Cu(acac)_2_ and Li-HMDS separately. From these experiments, no film growth was visibly detected for the Cu(acac)_2_ + TPD depositions, whereas the Li-HMDS + TPD depositions yielded relatively thin films that were air sensitive and unstable, reacting with air immediately after the exposure. These initial observations underlined the need to develop a three-precursor ALD/MLD process for the growth of the poly[Cu-ETT] films.

**Fig. 2:**
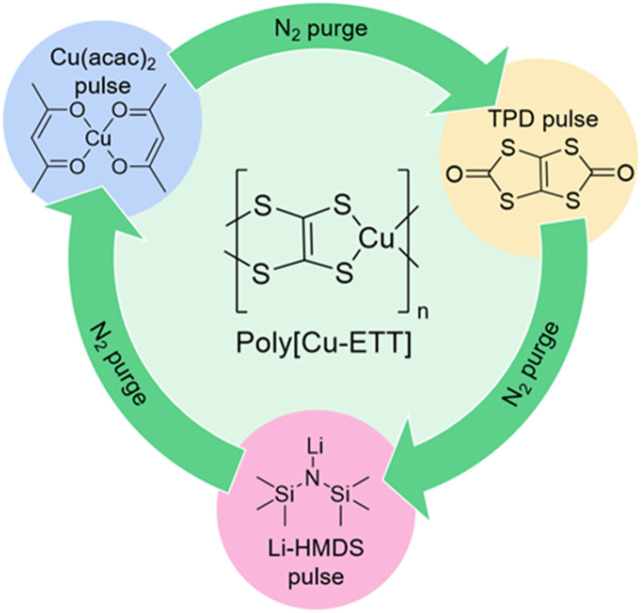
Three-precursor pulse/purge sequence of the ALD/MLD cycle used for the growth of the present copper–sulfur–organic thin films. After the initial surface activation with one Li-HMDS pulse, this three-precursor cycle starts from the TPD pulse.

In our three-precursor ALD/MLD process, all the depositions started with one Li-HMDS pulse to react with the OH-saturated surface of the silicon wafer. After this initial Li-HMDS pulse, the ALD/MLD cycle with the pulsing/purging sequence presented in Fig. 2 was applied, starting from the TPD pulse and N_2_ purge, followed by the Li-HMDS pulse and N_2_ purge, and completed with the Cu(acac)_2_ pulse and N_2_ purge. In this three-precursor ALD/MLD cycle, the Cu(acac)_2_/N_2_ step is responsible of the Cu-for-Li trans-metalation in the thin film and the removal of lithium as the Li(acac) byproduct.

**Fig. 3:**
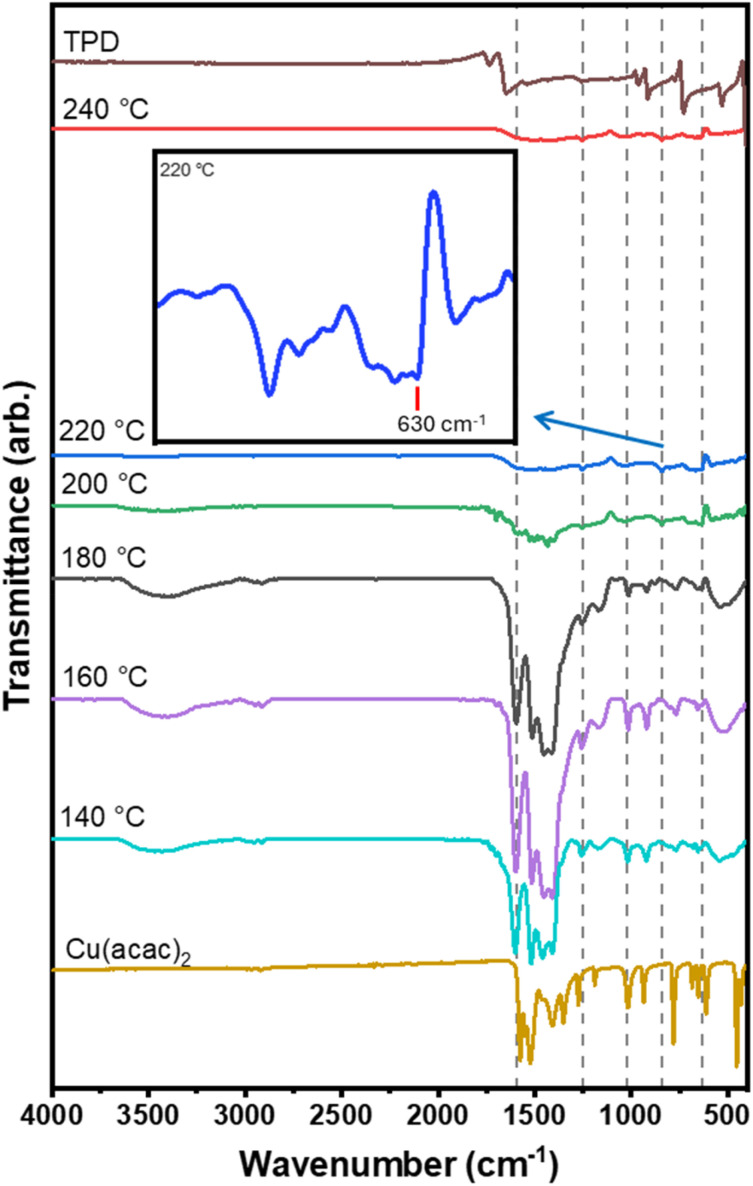
FTIR spectra for the copper–sulfur–organic polymer thin films deposited at different temperatures in the range of 140–240 °C; the IR-ATR spectra for the TPD and Cu(acac)_2_ precursors are also shown for reference. The inset shows a magnified low-frequency part of the film deposited at 220 °C; the peak indicated at 630 cm^−1^ is attributed to the Cu–S bond (the subtracted silicon peak around 600 cm^−1^ is the reason for the big drop in the intensity).

The initial tests to find the optimal deposition temperature range for the growth of the poly[Cu-ETT] films were carried out with 200 cycles and with a pulse length of 7 s for all three precursors and purge lengths of 30 s after the Li-HMDS and Cu(acac)_2_ pulses and 60 s after the TPD pulse. In Fig. 3 we display the FTIR spectra recorded for the films deposited in the temperature range of 140–240 °C. The strongest vibration peaks for films deposited at 140, 160 and 180 °C are around 1500 cm^−1^, corresponding to *ν*(C=O) at 1600–1650 cm^−1^, *ν*(C=C) at 1520 cm^−1^, and *ν*(C=H) at 1410 cm^−1^. These peaks stem from the remaining acetylacetonate ligands due to the incomplete reaction of the copper precursor. For these films, a wide peak around 3500 cm^−1^ belonging to O–H stretching is also seen; the most apparent source of these hydroxyl groups is the Li-HMDS precursor, which readily reacts with moisture to form (LiOH)_2_(HMDS)_2_. When the deposition temperature was increased to 200 °C the vibrations attributed to the acac ligands and hydroxyl groups were significantly decreased. The *ν*(C=O) peak at 1650 cm^−1^ was also negligible for the film grown at 220 °C, which suggests the successful opening of both carbonyl groups of the TPD molecule. For the films deposited below 200 °C, the peaks resemble those of the Cu(acac)_2_ and TPD precursors, and at 200 °C they are slightly shifted, which we interpret as an indication of some changes in the chemical environments of the TPD and acac units upon the polymer formation. The peaks due to the Cu(acac)_2_ precursor are no longer visible for the film deposited at 220 °C. For films deposited at 200 °C or higher, some new low-intensity features appear at wavenumbers between 620–650 cm^−1^, indicative of the formation of the Cu–S bonds in the poly[Cu-ETT] structure.^50–52^ These tiny features are best seen in the magnified close-up of the low wavenumber range of the FTIR spectrum of the 220 °C sample.

At this process development stage, we carried out XPS measurements for the four thin films deposited at 200, 210, 220 and 230 °C to especially estimate the Cu : Li ratio in the samples to be able to follow the progress of the trans-metalation reaction; the results are summarized in Table 1. It is seen that the Cu-for-Li trans-metalation is effective only for the films deposited at 220 °C or higher. Before fixing the deposition temperature for the rest of the experiments, we also carried out preliminary electrical conductivity measurements for our entire initial sample series (Fig. S1); it was found that the films deposited at 220 °C showed the highest electrical conductivities (and also appreciably high Seebeck coefficient values). Thus, this temperature was chosen as the deposition temperature for the rest of the experiments. Interestingly, regarding the electrical conductivity, our results suggest that it is beneficial that part of lithium remains in the polymer film as a counter-ion for the charge balance.

**Table 1:** XPS results for the Cu and Li contents (in atomic-%) in thin-film samples grown at different deposition temperatures (with 200 cycles)

Dep. temp. (°C)	Cu%	Li%	Cu : Li
230	6.6	1.0	6.6
220	4.6	1.7	2.8
210	5.9	7.1	0.8
200	6.2	6.6	0.9

**Fig. 4:**
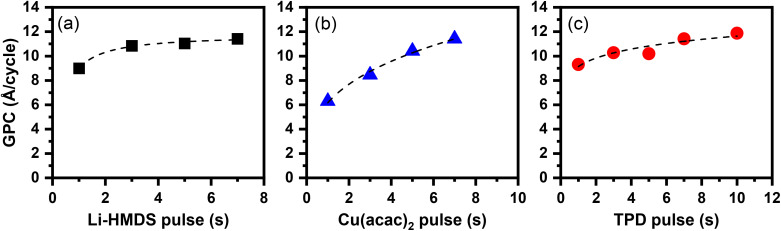
ALD/MLD process optimization data at a deposition temperature of 220 °C: increase/saturation of the GPC value with increasing (a) Li-HMDS pulse length, (b) TPD pulse length, and (c) Cu(acac)_2_ pulse length. While one of the precursor pulse lengths was varied the other two were fixed at 7 s; in each experiment the number of ALD/MLD cycles was 100.

The results of the deposition process optimization experiments carried out at a deposition temperature of 220 °C by applying 100 ALD/MLD cycles are summarized in Fig. 4. In these experiments the pulse length of one precursor was varied at a time while keeping the precursor pulse and purge lengths fixed for the other two precursors. The resultant film thicknesses were determined from the cross-sectional SEM images (Fig. S2); note that the “standard” XRR (X-ray reflection) and ellipsometry techniques most commonly employed for film thickness determination could not be used for the present samples due to the facts that the films were relatively thick and rough (making XRR modelling impossible) and possessed complex and deposition-parameter-dependent chemical compositions (making ellipsometry data modelling unreliable).

As shown in Fig. 4, the film growth clearly saturates regarding the Li-HMDS and TPD pulses, at 4 s and 7 s, respectively. For the Cu(acac)_2_ pulse, the complete saturation was not fully reached within the pulse length range studied (1–7 s), even though the curve shows some slow down effect for the longest pulses. Interestingly, with the Cu(acac)_2_ pulse length of 1 s (the Li-HMDS and TPD pulse lengths being at their saturated values), the resultant GPC value of ∼6 Å per cycle corresponds to the theoretical Cu–Cu distance in poly[Cu-ETT] (=6.08 Å). Then, the highest GPC value of ∼11 Å per cycle, seen on the Cu(acac)_2_ pulse length of 7 s implies that the polymer grows with an overall rate of approximately two [Cu-ETT] units in one ALD/MLD cycle, pointing towards a heavily crosslinked polymer structure.^53^ This might also emphasize the effect of trans-metalation on the GPC instead of classical saturative behaviour of ALD. It is also interesting to make a comparison with the ALD CuS thin films grown from Cu(acac)_2_ and elemental sulfur; for these thin films, a similarly high GPC value of ∼4 Å, corresponding to nearly twice the Cu–S bond length in CuS was seen.^54^ However, since the chosen deposition temperature of 220 °C is close to the decomposition temperature of Cu(acac)_2_, we should not totally ignore the possibility of partial decomposition of the copper precursor (causing CVD-type growth).^54–60^

The films were investigated with GIXRD measurements to rule out the possible formation of crystalline impurity phases such as Cu_2_S, and to confirm the amorphous nature of the poly[Cu-ETT] films (Fig. S3); note that Cu_2_S is highly conducting and could contribute positively to the electrical conductivity of the films. Four peaks at 2*θ* angles of 28.3°, 37.4° and 47.6° can be seen in the patterns. The peaks are very wide, implying the amorphous nature of our polymer films, similar to previous studies of poly[Ni-ETT] powders,^21^ but the peak positions are slightly different as the 3d-transition metal here is copper instead of nickel. As the deposition temperature increases to 240 °C, an additional peak appears at 32° and the peak at 47.6° shifts to lower angles, which could indicate the formation of traces of Cu_2_S impurity.^54^ The peaks from the silicon substrate are also overlapping with the wide and amorphous-like peak at 47.6°, and only clearly visible for the film deposited at 240 °C.

To address the elemental composition and to understand further the chemical bonding and redox schemes in our highest-quality Cu–S–organic thin film (grown at 220 °C), a detailed XPS analysis was conducted. The results revealed the presence of copper, sulfur and carbon, *i.e.* the main constituents of the intended polymer, as well as lithium left-over after the trans-metalation (apparently as a counter ion).^14,15^ Moreover, some (surface) impurities of oxygen, silicon and nitrogen were detected, see Table 2 and Fig. S4; for the Si and N impurities, the Li-HMDS or LiN(SiMe_3_)_2_ precursor is the apparent source. The assumed linear polymer structure of the poly[Cu-ETT] materials would result in a Cu : S ratio of 0.25.^14,31^ In our poly[Cu-ETT] thin-film sample, this ratio is 0.246, which is very close to the theoretical ratio. Theoretical ratios for copper and carbon and sulfur and carbon would be 0.50 and 2.00, respectively. From the XPS data these ratios were calculated as follows: Cu : C = 0.09 and S : C = 0.34, which are rather far from the theoretical values. This is due to the high amount of surface carbon, which has an overrated impact in the surface-sensitive XPS measurements. Another possible source of excess carbon could have been the accumulation of some acac ligands from the partially unreacted copper precursor, but according to the FTIR data this was considered negligible. The source of the detected nitrogen and silicon impurities is the Li-HMDS precursor.^59,60^

**Table 2:** XPS results for the elemental composition (atomic-%) of the poly[Cu-ETT] thin film grown at 220 °C with 200 cycles (with the optimized pulse/purge lengths)

Elements	Cu%	S%	C%	Li%	O%	Si%	N%
Cu-ETT	4.59	18.63	54.08	1.72	12.61	4.37	3.99

**Fig. 5:**
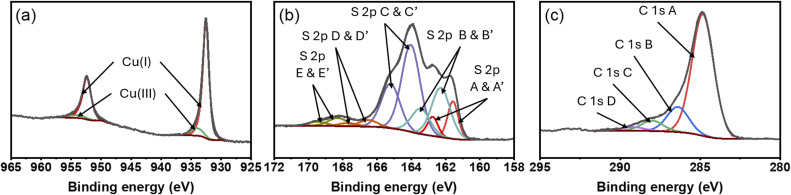
Core-level (a) copper 2p, (b) sulfur 2p and (c) carbon 1s XPS spectra for our poly[Cu-ETT] thin film grown at 220 °C with 200 cycles (with the optimized pulse/purge lengths).

In Fig. 5(a), the core-level Cu 2p XPS spectrum is shown to address the oxidation state of copper in our optimized poly[Cu-ETT] thin film. The spectrum exhibits sharp peaks for the Cu 2p_3/2_ and Cu 2p_1/2_ doublet, with the 2p_3/2_ peak located at approximately 932.5 eV; this is typical for the Cu(i) oxidation state. Additionally, the spectrum contains a smaller and broader component located at approximately 934.0 eV due to higher valent copper. The Cu(i) component is significantly larger of the two, the relative concentrations being 90.6% for Cu(i) and 9.4% for the higher valent copper. The minor component could be assigned to either Cu(ii) or Cu(iii) based on previous relevant literature.^14,18,53^ From the common chemistry knowledge, the high-valent Cu(iii) is most readily stabilized only when copper is bonded to the most electronegative anions (O or F), and hence its presence is unlikely in the current Cu–S bonding scheme. Also the coexistence of Cu(iii) with Cu(i) is unusual. On the other hand, for Cu(ii) we typically expect to see a smaller satellite peak around ∼940 eV; this was not detected for our sample. It is of course possible that the Cu(ii) content is just so small that the satellite is not visible.^61,62^ We also collected the Cu LMM Auger spectrum for the same sample (see Fig. S5) to support the aforementioned assignments and to rule out the metallic copper. Overall, an interesting point to note is the fact that the divalent copper precursor yields thin films in which monovalent copper dominates. This resembles the reported Cu(acac)_2_ + H_2_O ALD process, which yields Cu_2_O films.^56^

In Fig. 5(b), the S 2p core-level spectrum of sulfur is presented. Most of the sulfur atoms belong to components C and B with 2p_3/2_ binding energies of 162.3 eV and 164.1 eV, respectively; these are believed to belong to the ETT/TTO polymer.^14,18,53^ The copper sulfide-like bonding is present as the small components A and A′ with 2p_3/2_ binding energies of 161.6 eV and 162.8 eV, respectively. The full-width-at-half-maximum for the A and A′ peaks is smaller in comparison to the B and C components, which may be caused by differences in material properties, such as conductivity or crystallinity of these bonding modes.^63^ Earlier studies on different poly[M-ETT/TTO] bulk samples have revealed that the conduction mechanism is most likely hopping transport, and the organic backbone of the polymer barely contributes to the conductivity.^15^ The D and E components are presumably related to surface oxidation and impurities with different oxidation states of sulfur.^15,18,53^

The C 1s spectrum is presented in Fig. 5(c) and fitted with four Gaussian–Lorentzian peaks for the standard components A, B, C and D. The presence of adventitious carbon on these samples prevents us from carrying out in depth analysis of the C 1s spectrum. The expected energies from the ethylene tetrathiolate compound would probably be located close to the energy for aliphatic carbon at 284.8 eV (C–C) or slightly shifted toward the energy of the C–O component at 286.5 eV.^15,31^ No additional peaks were needed for a good fitting of the spectrum, but this does not necessarily mean that a C–S component is not present in the sample. The difference between the carbon spectrum of poly[Ni-ETT] and poly[Ni-TTO] has been studied, and the biggest differences were found for the D component at 287.8 eV,^15^ which in our case is small, thus indicating that the majority of the polymer is poly[Cu-ETT].

**Fig. 6:**
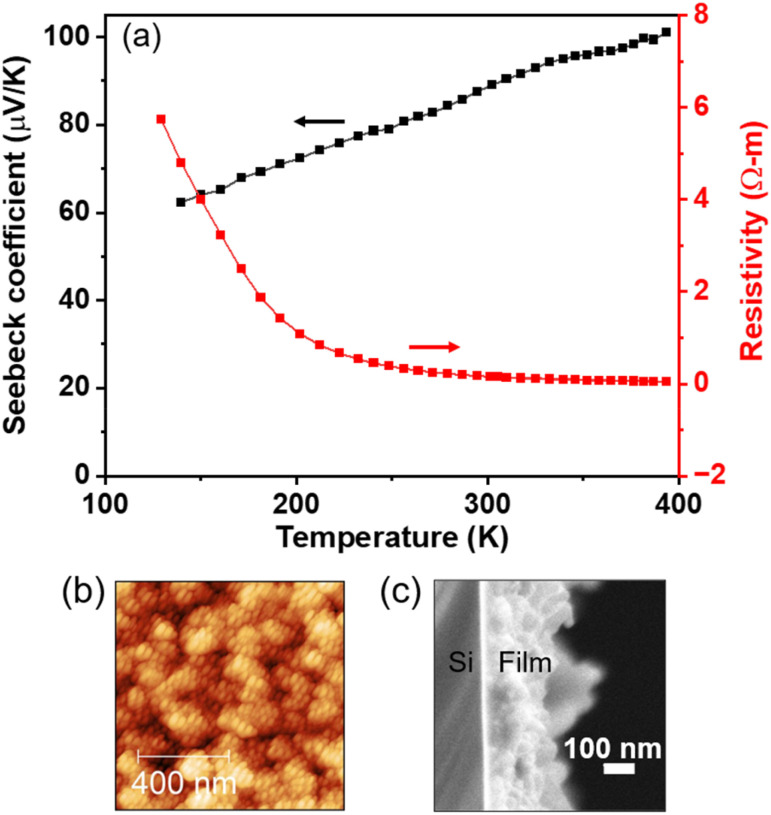
(a) Temperature dependence of electrical resistivity and the Seebeck coefficient, (b) AFM image, and (c) cross-sectional SEM image for our poly[Cu-ETT] thin films grown at 220 °C with 200 cycles (with the optimized pulse/purge lengths). In (a) and (c) the number of deposition cycles was 200, while in (b) it was 100.

The electrical transport data (resistivity and Seebeck coefficient) measured in the temperature range of 120–400 K for the poly[Cu-ETT] thin film deposited with 200 cycles (∼220 nm) at 220 °C are displayed in Fig. 6(a). The temperature behavior, *i.e.* increasing resistivity with decreasing temperature, indicates that the material is a semiconductor, while the positive Seebeck coefficient confirms the expected p-type conductivity. The 300-K values for electrical resistivity and the Seebeck coefficient are 0.17 Ω m^−1^ and 88 µV K^−1^, respectively. The Seebeck coefficient is slightly higher compared to the values of 80, 54.8 and 50.5 µV K^−1^ reported for powder samples.^14,18,52^ Electrical conductivity of our poly[Cu-ETT] films (*σ* = 0.06 S cm^−1^) is rather low compared to the reported 300-K values of 1.03, 0.76, 88.6, 59.4 and 2.5–42.0 S cm^−1^.^14,18,27,53^ The reported electrical conductivities and Seebeck coefficients of the polymer powders vary depending on the metal salt, alkali metal counter-ion and oxidation conditions applied during the synthesis, which explains the variation seen.

The stability of electrical conductivity in poly[M-ETT] materials has been studied over the years and there are several studies reporting concerns for the stability of n-type poly[Ni-ETT] and poly[Ni-TTO] materials.^15^ We also observed some decrease in electrical conductivity after storing the film for couple of days under ambient conditions. In our future studies, we aim to tackle this issue by *in situ* depositing a thin barrier layer to protect our poly[Cu-ETT] films from ambient conditions.^64^

## Conclusions

We have demonstrated the fabrication of electrically conductive metal–organic coordination polymer thin films by ALD/MLD. The three-precursor ALD/MLD process developed is rather unique as such. Also, among the three precursors employed, the organic precursor TPD, which contains the carbon–sulfur backbone of the polymer, has not been previously used in the context of MLD or ALD/MLD. This precursor does not directly react with the copper precursor Cu(acac)_2_; hence an activation step by Li-HMDS is needed to remove the carbonyl group from TPD. After this activation step, the trans-metalation with Cu(acac)_2_ readily occurs.

For the efficient Cu-for-Li trans-metalation process to occur, a deposition temperature of 220 °C or higher is needed. The optimal deposition temperature range may be relatively narrow, as at temperatures higher than 230 °C the decomposition of the Cu(acac)_2_ precursor may become an issue. For the poly[Cu-ETT] thin films deposited at 220 °C XPS analysis confirmed the presence of copper and sulfur in an essentially ideal ratio of 0.246. At the same time, the Cu : Li ratio was estimated to be *ca.* 2.8; we believe the lithium remaining after the trans-metalation acts as a counter ion in the polymer network, thereby controlling the redox state of copper and possibly also the thermoelectric characteristics.

The poly[Cu-ETT] films deposited at 220 °C were confirmed to be p-type semiconductors, showing electrical transport properties relevant for application in thermoelectrics: a resistivity of 0.17 Ω-m and a Seebeck coefficient of 88 µV K^−1^ at 300 K. These values align well with those reported in previous studies for powder poly[Cu-ETT] samples. The somewhat low electrical conductivity (together with the high Seebeck value) can stem from the fact that the films were not yet fully optimized for the TE properties; we assume that this would require better tuning of the oxidation state of copper/amount of counter-ion lithium, which will be addressed in our future studies.

We believe that the ALD/MLD method has clear advantages as a fabrication technology for future wearable thermoelectric applications requiring – besides feasible TE characteristics – mechanical flexibility and conformality for efficient textile integration.

## Author contributions

All authors contributed to the study and have given approval to the final version of the manuscript. Mari Heikkinen: conceptualization, methodology, investigation, visualization, writing – original draft. Malar Auxilia Francis: conceptualization, investigation, writing – review & editing. Kristoffer Meinander: methodology, investigation, writing – review & editing. Girish C. Tewari: investigation. Mikko Nisula: validation. Maarit Karppinen: conceptualization, supervision, funding acquisition, writing – review & editing.

## Conflicts of interest

There are no conflicts to declare.

## Supplementary Material

TA-014-D6TA01174H-s001

## Data Availability

The data supporting this article have been included as part of the supplementary information (SI). Supplementary information is available. See DOI: https://doi.org/10.1039/d6ta01174h.

## References

[cit1] Jia Y., Jiang Q., Sun H., Liu P., Hu D., Pei Y., Liu W., Crispin X., Fabiano S., Ma Y., Cao Y. (2021). Adv. Mater..

[cit2] Prunet G., Pawula F., Fleury G., Cloutet E., Robinson A. J., Hadziioannou G., Pakdel A. (2021). Mater. Today Phys..

[cit3] Zhu S., Miao L., Gao J., liang Chen J., Zhou Q., Pan Z., Zhang Z., Liang J., Yang X., Mori T. (2025). Nano Energy.

[cit4] Miao L., Zhu S., Liu C., Gao J., Zhang Z., Peng Y., Chen J. L., Gao Y., Liang J., Mori T. (2024). Nat. Commun..

[cit5] Soumya S., Fatima K. S., Lekshmi S., Namboothiri S. G., Krishnapriya P. K., Shreya V. A., Harikrishnan V. S., Mohan A. C., Joh H., Rani J. R., Panwar V., Sreedhar K. M., P J., Samanta S., Jo J. Y., Anoop G. (2025). J. Alloys Compd..

[cit6] Wan C., Gu X., Dang F., Itoh T., Wang Y., Sasaki H., Kondo M., Koga K., Yabuki K., Snyder G. J., Yang R., Koumoto K. (2015). Nat. Mater..

[cit7] Wan C., Wang Y., Wang N., Norimatsu W., Kusunoki M., Koumoto K. (2010). Sci. Technol. Adv. Mater..

[cit8] Liu J., Liu Q., Lin S., Leung M. Y., Ma Y., Tao X. (2023). Phys. Status Solidi RRL.

[cit9] Paleti S. H. K., Kim Y., Kimpel J., Craighero M., Haraguchi S., Müller C. (2024). Chem. Soc. Rev..

[cit10] Lindorf M., Mazzio K. A., Pflaum J., Nielsch K., Brütting W., Albrecht M. (2020). J. Mater. Chem. A.

[cit11] Niemelä J. P., Karttunen A. J., Karppinen M. (2015). J. Mater. Chem. C.

[cit12] Wu W. N., Zheng Q. B., Liu C. L. (2024). Synth. Met..

[cit13] Nisula M., Karttunen A. J., Solano E., Tewari G. C., Karppinen M., Minjauw M., Jena H. S., Van Der Voort P., Poelman D., Detavernier C. (2021). ACS Appl. Mater. Interfaces.

[cit14] Sun Y., Sheng P., Di C., Jiao F., Xu W., Qiu D., Zhu D. (2012). Adv. Mater..

[cit15] Tkachov R., Stepien L., Grafe R., Guskova O., Kiriy A., Simon F., Reith H., Nielsch K., Schierning G., Kasinathan D., Leyens C. (2018). Polym. Chem..

[cit16] Wolfe R. M. W., Menon A. K., Marder S. R., Reynolds J. R., Yee S. K. (2019). Adv. Electron. Mater..

[cit17] Menon A. K., Uzunlar E., Wolfe R. M. W., Reynolds J. R., Marder S. R., Yee S. K. (2017). J. Appl. Polym. Sci..

[cit18] Sheng P., Sun Y., Jiao F., Di C., Xu W., Zhu D. (2014). Synth. Met..

[cit19] Oshima K., Shiraishi Y., Toshima N. (2015). Chem. Lett..

[cit20] Menon A. K., Meek O., Eng A. J., Yee S. K. (2017). J. Appl. Polym. Sci..

[cit21] Sun Y., Qiu L., Tang L., Geng H., Wang H., Zhang F., Huang D., Xu W., Yue P., Guan Y. S., Jiao F., Sun Y., Tang D., Di C. A., Yi Y., Zhu D. (2016). Adv. Mater..

[cit22] Sun Y., Zhang J., Liu L., Qin Y., Sun Y., Xu W., Zhu D. (2016). Sci. China Chem..

[cit23] Liu L., Sun Y., Li W., Zhang J., Huang X., Chen Z., Sun Y., Di C., Xu W., Zhu D. (2017). Mater. Chem. Front..

[cit24] Liu Z., Liu T., Savory C. N., Jurado J. P., Reparaz J. S., Li J., Pan L., Faul C. F. J., Parkin I. P., Sankar G., Matsuishi S., Campoy-Quiles M., Scanlon D. O., Zwijnenburg M. A., Fenwick O., Schroeder B. C. (2020). Adv. Funct. Mater..

[cit25] Wang Y., Yao Q., Qu S., Chen L. (2022). J. Wuhan Univ. Technol.-Mater. Sci. Ed..

[cit26] Yura R., Itoh T., Ishihara M., Suzuki D., Kuo Y.-C., Nagata S., Yumura T., Hosokawa S., Murata M., Nonoguchi Y. (2025). J. Mater. Chem. A.

[cit27] Holdcroft G. E., Underhill A. E. (1985). Synth. Met..

[cit28] Vicente R., Ribas J., Cassoux P., Valade L. (1986). Synth. Met..

[cit29] Faulmann C., Cassoux P., Vlcente R., Ribas J., Jolly C. A., Reynolds J. R. (1989). Synth. Met..

[cit30] Tkachov R., Stepien L., Roch A., Komber H., Hennersdorf F., Weigand J. J., Bauer I., Kiriy A., Leyens C. (2017). Tetrahedron.

[cit31] Menon A. K., Wolfe R. M. W., Marder S. R., Reynolds J. R., Yee S. K. (2018). Adv. Funct. Mater..

[cit32] Hwang S., Potscavage W. J., Kim T. W., Adachi C. (2020). Adv. Electron. Mater..

[cit33] Hu Y., Rivers G., Weir M. P., Amabilino D. B., Tuck C. J., Wildman R. D., Makarovsky O., Woodward S. (2024). Electr. Mater. Lett..

[cit34] Ueda K., Yamada Y., Terao T., Manabe K., Hirai T., Asaumi Y., Fujii S., Kawano S., Muraoka M., Murata M. (2020). J. Mater. Chem. A.

[cit35] Multia J., Karppinen M. (2022). Adv. Mater. Interfaces.

[cit36] Vazquez-Arce J. L., Shin D. H., Yao S., He S., Nielsch K., Bahrami A. (2025). Adv. Energy Mater..

[cit37] Sarnet T., Hatanpää T., Puukilainen E., Mattinen M., Vehkamäki M., Mizohata K., Ritala M., Leskelä M. (2015). J. Phys. Chem. A.

[cit38] Yang J., Mukherjee S., Lehmann S., Krahl F., Wang X., Potapov P., Lubk A., Ritschel T., Geck J., Nielsch K. (2023). Small.

[cit39] Yang J., Daqiqshirazi M., Ritschel T., Bahrami A., Lehmann S., Wolf D., Feng W., Pöhl A., Charvot J., Bureš F., Brumme T., Lubk A., Geck J., Nielsch K. (2024). ACS Nano.

[cit40] Cremers V., Puurunen R. L., Dendooven J. (2019). Appl. Phys. Rev..

[cit41] Heikkinen M., Ghiyasi R., Karppinen M. (2025). Adv. Mater. Interfaces.

[cit42] Ruoho M., Tarasiuk N., Rohbeck N., Kapusta C., Michler J., Utke I. (2018). Mater. Today Chem..

[cit43] Philip A., Niemelä J. P., Tewari G. C., Putz B., Edwards T. E. J., Itoh M., Utke I., Karppinen M. (2020). ACS Appl. Mater. Interfaces.

[cit44] Tynell T., Terasaki I., Yamauchi H., Karppinen M. (2013). J. Mater. Chem. A.

[cit45] Krahl F., Giri A., Tomko J. A., Tynell T., Hopkins P. E., Karppinen M. (2018). Adv. Mater. Interfaces.

[cit46] Karttunen A. J., Sarnes L., Townsend R., Mikkonen J., Karppinen M. (2017). Adv. Electron. Mater..

[cit47] Marin G., Funahashi R., Karppinen M. (2020). Adv. Eng. Mater..

[cit48] Madadi M., Heiska J., Multia J., Karppinen M. (2021). ACS Appl. Mater. Interfaces.

[cit49] Giedraityte Z., Sainio J., Hagen D., Karppinen M. (2017). J. Phys. Chem. C.

[cit50] Raj S. I., Jaiswal A., Uddin I. (2020). RSC Adv..

[cit51] Pei L. Z., Wang J. F., Tao X. X., Wang S. B., Dong Y. P., Fan C. G., Zhang Q.-F. (2011). Mater. Charact..

[cit52] Raj S. I., Jaiswal A. (2021). J. Photochem. Photobiol. A-Chem..

[cit53] Sheng P., Sun Y., Jiao F., Liu C., Xu W., Zhu D. (2014). Synth. Met..

[cit54] Tripathi T. S., Lahtinen J., Karppinen M. (2018). Adv. Mater. Interfaces.

[cit55] Hu X., Schuster J., Schulz S. E., Gessner T. (2015). Phys. Chem. Chem. Phys..

[cit56] Bartholazzi G., Shehata M. M., Macdonald D. H., Black L. E. (2023). J. Vac. Sci. Technol. A.

[cit57] Utriainen M., Kröger-Laukkanen M., Johansson L.-S., Niinistö L. (2000). Appl. Surf. Sci..

[cit58] Alnes M. E., Monakhov E., Fjellvåg H., Nilsen O. (2012). Chem. Vap. Deposition.

[cit59] Haukka S., Root A. (1994). J. Phys. Chem..

[cit60] Pieters M. J., Bartel L., van Helvoirt C., Creatore M. (2024). J. Phys. Chem. C.

[cit61] Biesinger M. C., Lau L. W. M., Gerson A. R., Smart R. S. C. (2010). Appl. Surf. Sci..

[cit62] Biesinger M. C. (2017). Surf. Interface Anal..

[cit63] Beamson G., Clark D. T., Hayes N. W., Law D. S.-L. (1994). Surface Science Spectra.

[cit64] Jussila T., Pekkanen J., Virta A., Ghazy A., Lastusaari M., Karppinen M. (2025). J. Vac. Sci. Technol. A.

