# Supplementary material for: Atomic/molecular layer deposition of p-type conducting copper–sulfur–organic coordination polymer thin films for thermoelectric applications
Source: J Mater Chem A Mater. 2026 Mar 11;14(27):17229–36. doi: 10.1039/d6ta01174h (PMC13015183; doi:10.1039/d6ta01174h)
Supplement: TA-014-D6TA01174H-s001 [file TA-014-D6TA01174H-s001.pdf]

Supplementary Information (SI) for Journal of Materials Chemistry A.
This journal is © The Royal Society of Chemistry 2026

Supporting information:

Atomic/molecular layer deposition of p-type conducting copper-sulfur-organic coordination polymer thin films for thermoelectric applications

*Mari Heikkinen[a], Francis Malar Auxilia[a], Kristoffer Meinander[b], Girish C. Tewari[a], Mikko Nisula[a], Maarit Karppinen[a]**

[a] Department of Chemistry and Materials Science, Aalto University, FI-00076 Espoo, Finland

[b] Department of Bioproducts and Biosystems, Aalto University, FI-00076 Espoo, Finland

E-mail: maarit.karppinen@aalto.fi

Table S1. Comprehensive list of different $M'_x[M''\text{-ETT}]$ polymer materials by the center metal and opening reactant (base) used in their synthesis with electrical conductivity, type of charge carrier and type of end product. If the conductivity was not measured at room temperature the temperature is given with conductivity.

Metal	Material	Base	Conductivity S/cm (RT)	Charge carrier	Powder or film	Year and reference
**Fe**	M'_x[Fe-ETT]	N/A	0.3	-	Powder	1997 [1]
**Pt**	M'_x[Pt-ETT]	NaOEt	3.1	-	Powder	1985 [2]
	Na_x[Pt-ETT]	NaOMe	1.9	-	Powder	1985 [3]
**Pd**	Na_x[M-ETT]	NaOEt	Insulator	-	Powder	1985 [2]
	Na_x[M-ETT]	NaOMe	6.4×10^{-2}	-	Powder	1985 [3]
**Ni**	Na_x[Ni-ETT]	NaOEt	2.4×10^{-5}–5.4	-	Powder	1985 [2]
	Na_x[Ni-ETT]	NaOMe	3.8	-	Powder	1985 [3]
	M'_x[Ni-ETT]	N/A	not given	-	Powder	1989 [4]
	M'_x[Ni-ETT]	N/A	0.5	-	Powder	1997 [1]
	Na_x[Ni-ETT]	NaOMe	40	n-type	Powder	2012 [5]
	K_x[Ni-ETT]	KOMe	44	n-type	Powder	2012 [5]
	Ni_x[Ni-ETT]	NaOMe	6.6	n-type	Powder	2012 [5]
	M'_x[Ni-ETT]	N/A	1.14–1.55	n-type	Powder	2015 [6]
	K_x[Ni-ETT] /PVDF/DMSO	KOMe	0.5–43.0	n-type	Film	2017 [7,8]
	M'_x[Ni-ETT]	KOMe	38–54, 209–	n-type	Film	2016 [9,10]

			227			
	K_x[Ni-ETT]	KOMe	192.5–252.5	n-type	Film	2017 [11]
	Na_x[Ni_x-ETT]/PVDF	NaOMe	50	n-type	Film	2018 [12]
	Na_x[Ni-ETT]/PVDF	NaOMe	6–23	n-type	Film	2018 [13]
	Ni-TTO	KOMe	27–47	n-type	Powder	2017, 2018 [14,15]
	K_x[Ni-ETT]	KOMe	24.3	n-type	Powder	2017, 2018 [14,15]
	Ni-TTO/PVDF	N/A	18.2	n-type	Powder	2019 [16]
	Na_x[Ni-ETT]	NaOMe	150	n-type	Film	2020 [17]
	Na_x[Ni-ETT]	NaOMe	10	n-type	Powder	2020 [18]
	Na_x[Ni-ETT]	NaOMe	39	n-type	Film	2020 [19]
	Na_x[Ni-ETT]	NaOMe	17–50	n-type	Powder	2022 [20]
	$(EtCO_2Ba)_x$[Ni-ETT]	$(EtCO_2)$BaOH	3.7x10^{-2}	n-type	Powder	2024 [21]
	Na_x[Ni-ETT]	NaOMe	23.7	-	Powder /film	2025 [22]
	Na_x[Ni-ETT] /CNT/PVC	NaOMe	429 & 548/340 K	p-type	Film	2015, 2020 [23,24]
**Co**	M'_x[Co-ETT]	Na	4.0x10^{-4}/ Insulator	-	Powder	1985 [2]
	M'_x[Co-ETT]	N/A	0.3	-	Powder	1997 [1]
**Cr**	M'_x[Cr-ETT]	N/A	1.0x10^{-3}	-	Powder	1997 [1]
**Cu**	M'_x[Cu-ETT]	Na	2.9–42.0/ Insulator	-	Powder	1985 [2]
	M'_x[Cu-ETT]	NaOH	27.7	-	Powder	1985 [2]
	M'_x[Cu-ETT]	KOH	5.0	-	Powder	1985 [2]
	$(NEt_4)_x$[Cu-ETT]	N/A	not given	-	Powder	1988 [4]
	M'_x[Cu-ETT]	N/A	<1.0x10^{-3}	-	Powder	1997 [1]
	Cu_x[Cu-ETT]	NaOMe	9.5	p-type	Powder	2012 [5]
	Na_x[Cu-ETT]	NaOMe	0.2	p-type	Powder	2012 [5]
	Cu_x[Cu-ETT]	NaOH	10.7 & 88.6	p-type	Powder	2014 [25,26]
**Zn**	M'_x[Zn-ETT]	N/A	<1.0x10^{-3}	-	Powder	1997 [1]
**Mn**	Na_x[Mn-ETT]	Na	Insulator	-	Powder	1985 [2]
**Au**	Na_x[Au-ETT]	NaOMe	3.3	-	Powder	1985 [3]
**Hg**	Hg-ETT	N/A	0.02	-	Powder	1997 [1]

* M'_x denotes the counterion and N/A denotes the base, in cases when they are left unidentified. Insulator means conductivity <10^{-8} S/cm.

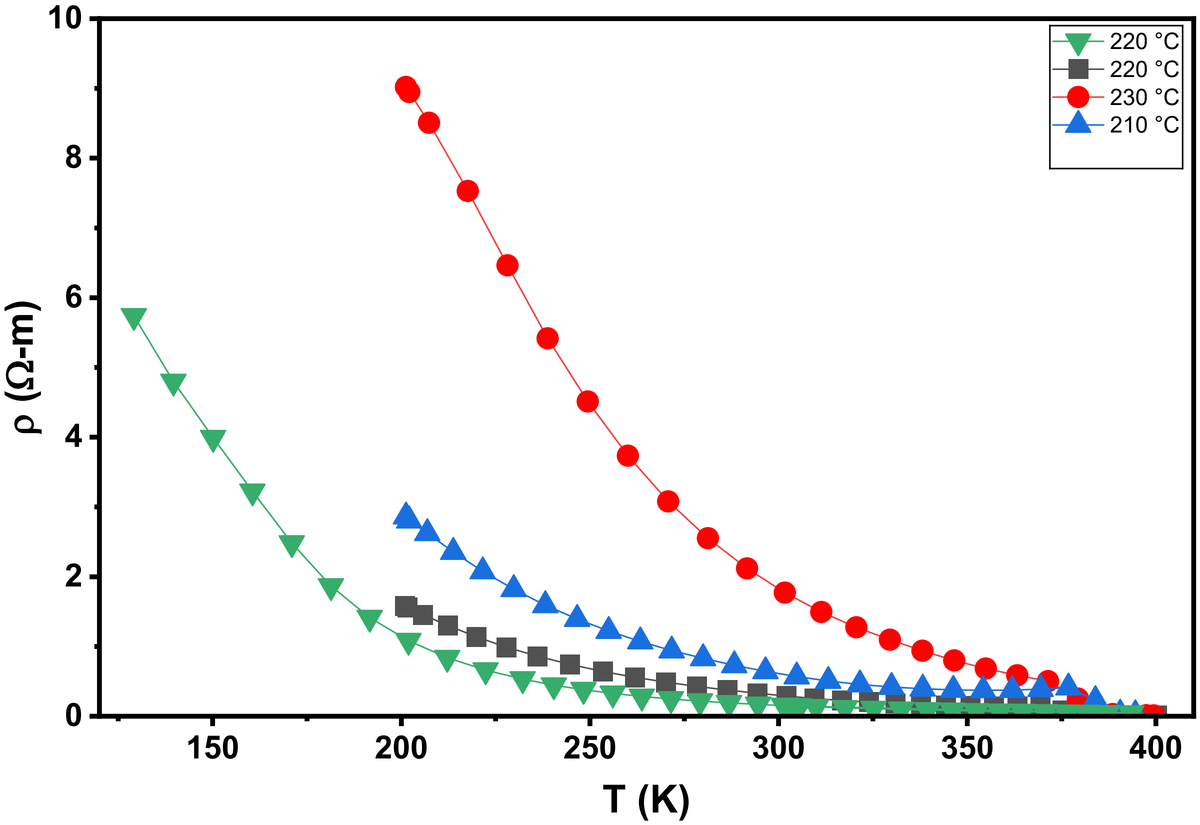

Figure S1. Temperature dependence of electrical resistivity measured for poly[Cu-ETT] thin films deposited at 210, 220 (two parallel samples) and 230 °C.

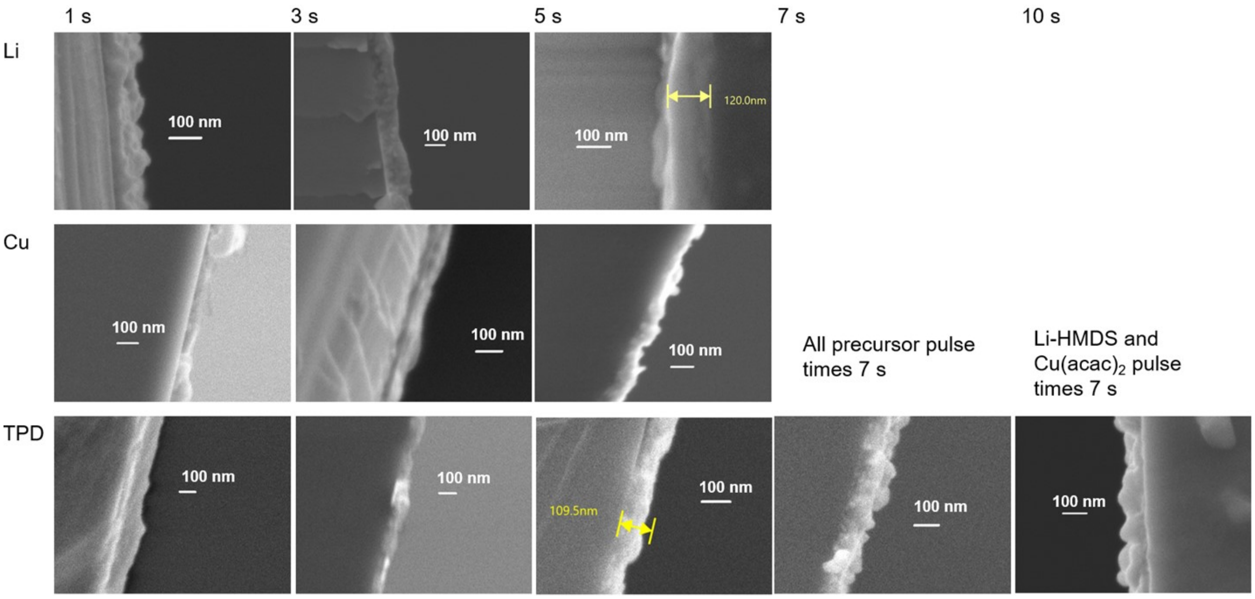

Figure S2. Cross sectional SEM images for poly[Cu-ETT] thin films deposited at 220 °C using different precursor pulse times; these images were collected for the sample series deposited for the investigation

of the surface saturation behavior. The film thickness value was taken as an average of thicknesses measured for the thinnest point, for the thickest point and for some points of the film.

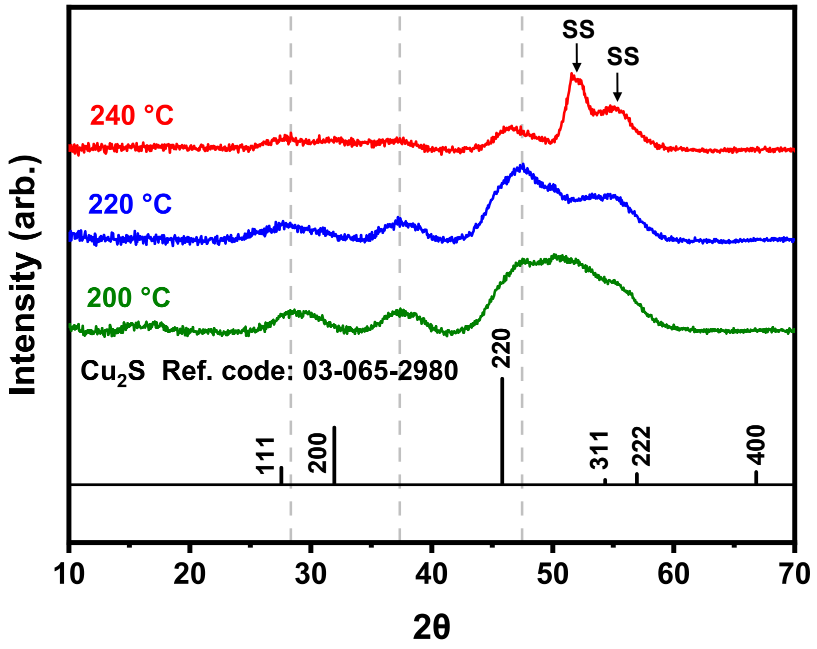

Figure S3. GIXRD patterns for poly[Cu-ETT] thin films deposited at different temperatures (200, 220 or 240 °C) with 200 cycles; peaks due to the silicon substrate are denoted as SS.

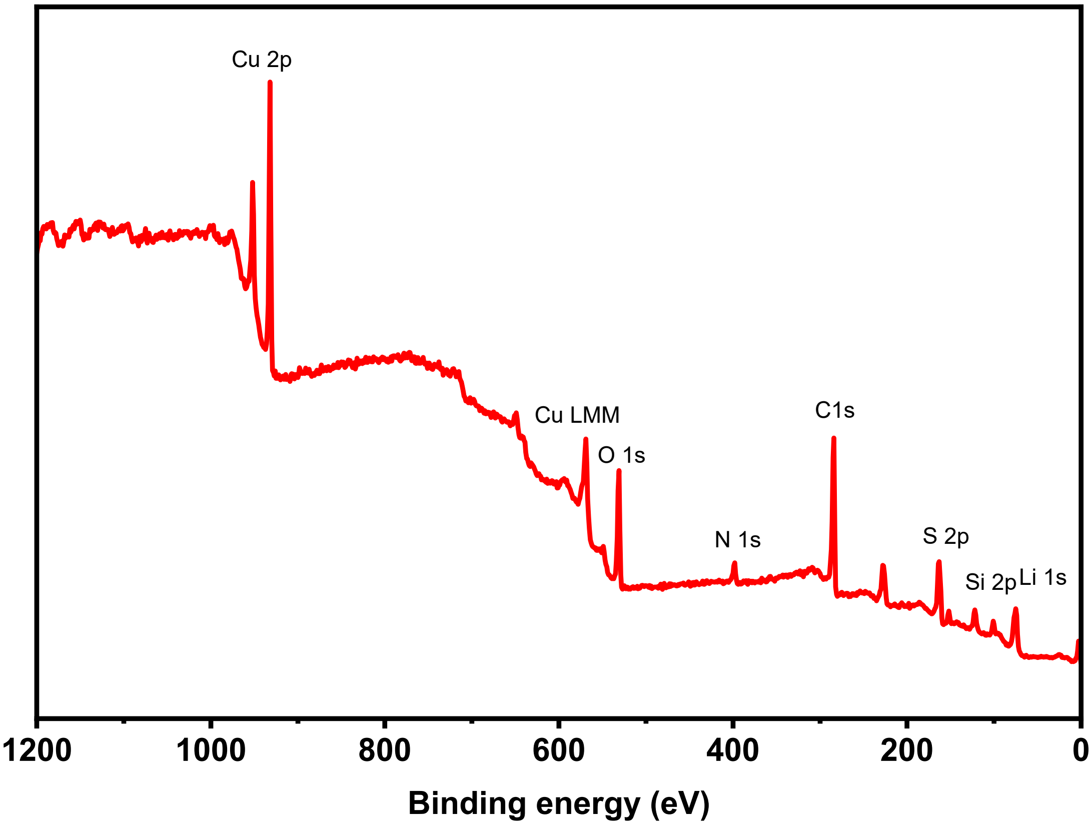

Figure S4. XPS survey spectrum for a poly[Cu-ETT] thin film deposited at 220 °C.

Presence of nitrogen in the survey spectrum was addressed as follows: two distinct N 1s components were clearly visible, which could be fitted with two Gaussian-Lorentzian components, located at approximately 398.7 eV and 400.6 eV. The lower binding energy is similar to what could be expected for Si-N bonding e.g. in trisilylamine, and most likely originates from nitrogen in unreacted Li-HMDS precursor. The higher binding energy is typical for C-N bonding in amines. Most of the intensity (76.6%) in the N 1s spectra was found to be in the lower binding energy component.

Presence of silicon in the survey spectrum was addressed as follows: the Si 2p component was fitted with a single Gaussian-Lorentzian peak at approximately 101.5 eV, which is a typical energy for Si-N bonding, most likely related to the unreacted Li-HMDS precursor. This energy, although slightly low, might however also be present for Si-O bonding in silicon oxides.

Presence of lithium in the survey spectrum was addressed as follows: the Li 1s component was fitted with a single peak at approximately 55.7 eV. Typically, there is not a large shift in binding energy for different compounds of lithium, and the measured binding energy can be associated with several different sources of ionic lithium.

A binding energy of 932.5 eV of the 2p3/2 peak is typical for both Cu(I) metallic Cu. To distinguish between these two copper states, Cu LMM Auger spectrum was also collected, see below. The strong peak seen at ca. 569.4 eV translates to a kinetic energy of 917.2 eV, and a modified Auger parameter of 1849.7 eV. These values are typical for Cu(I) in copper-sulfur bonding. For metallic copper, a peak around 918.8 eV, as well as sharp secondary features, would have been expected but these are not seen in the recorded spectrum, effectively excluding the possibility for metallic copper in the sample.

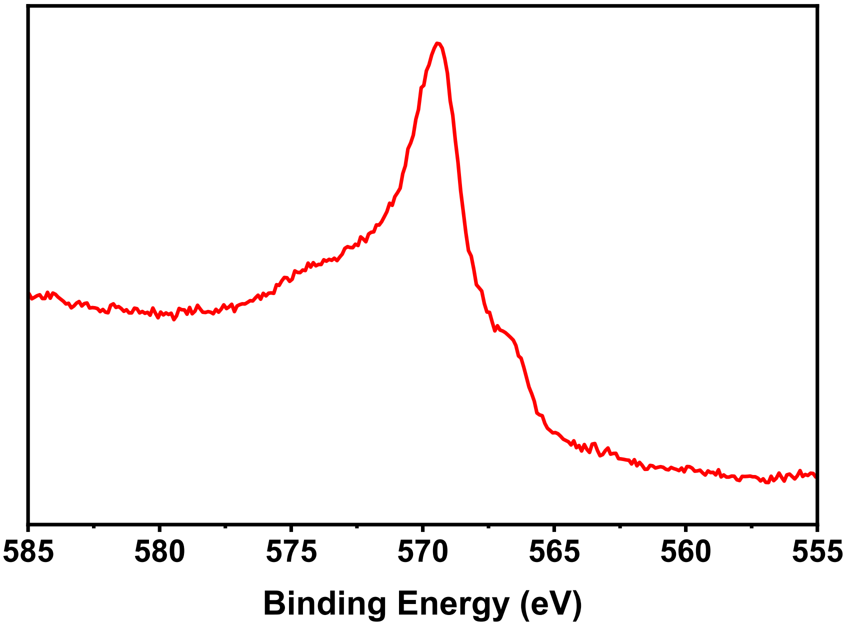

Figure S5. XPS Cu LMM Auger spectrum for a poly[Cu-ETT] thin film deposited at 220 ^{o}C.

REFERENCES

1 D. G. Xenikos, H. Müller, C. Jouan, A. Sulpice and J. L. Tholenceb, *Solid State Communications,* 1997, **102**, 681-685.

2 G. E. Holdcroft and A. E. Underhill, *Synth. Met.*, 1985, **10**, 427–434.

3 R. Vicente, J. Ribas, P. Cassoux and L. Valade, *Synth. Met.*, 1986, **13**, 265–280.

4 C. Faulmann, P. Cassoux, R. Vlcente, J. Ribas, C. A. Jolly and J. R. Reynolds, *Synth. Met.,* 1989, **29**, E557-E562.

5 Y. Sun, P. Sheng, C. Di, F. Jiao, W. Xu, D. Qiu and D. Zhu, *Advanced Materials*, 2012, **24**, 932–937.

6 K. Oshima, Y. Shiraishi and N. Toshima, *Chem. Lett.*, 2015, **44**, 1185–1187.

7 A. K. Menon, O. Meek, A. J. Eng and S. K. Yee*, J. Appl. Polym. Sci*., 2017, **134**, 44060.

8 A. K. Menon, E. Uzunlar, R. M. W. Wolfe, J. R. Reynolds, S. R. Marder and S. K. Yee, *J. Appl. Polym. Sci.*, 2017, **134**, 44402.

9 Y. Sun, L. Qiu, L. Tang, H. Geng, H. Wang, F. Zhang, D. Huang, W. Xu, P. Yue, Y. S. Guan, F. Jiao, Y. Sun, D. Tang, C. A. Di, Y. Yi and D. Zhu, *Advanced Materials*, 2016, **28**, 3351–3358.

10 Y. Sun, J. Zhang, L. Liu, Y. Qin, Y. Sun, W. Xu and D. Zhu, *Sci. China Chem.*, 2016, **59**, 1323–1329.

11 L. Liu, Y. Sun, W. Li, J. Zhang, X. Huang, Z. Chen, Y. Sun, C. Di, W. Xu and D. Zhu, *Mater. Chem. Front.*, 2017, **1**, 2111–2116.

12 A. K. Menon, R. M. W. Wolfe, S. R. Marder, J. R. Reynolds and S. K. Yee, *Adv. Funct. Mater.*, DOI:10.1002/adfm.201801620.

13 R. M. W. Wolfe, A. K. Menon, T. R. Fletcher, S. R. Marder, J. R. Reynolds and S. K. Yee, *Adv. Funct. Mater.*, 2018, **28**, 1801620.

14 R. Tkachov, L. Stepien, A. Roch, H. Komber, F. Hennersdorf, J. J. Weigand, I. Bauer, A. Kiriy and C. Leyens, *Tetrahedron*, 2017, **73**, 2250–2254.

15 R. Tkachov, L. Stepien, R. Grafe, O. Guskova, A. Kiriy, F. Simon, H. Reith, K. Nielsch, G. Schierning, D. Kasinathan and C. Leyens, *Polym. Chem.*, 2018, **9**, 4543–4555.

16 R. M. W. Wolfe, A. K. Menon, S. R. Marder, J. R. Reynolds and S. K. Yee, *Adv. Electron. Mater.*, 2019, **5**, 1900066.

17 S. Hwang, W. J. Potscavage, T. W. Kim and C. Adachi, *Adv. Electron. Mater.*, 2020, **6**, 1901172.

18 Z. Liu, T. Liu, C. N. Savory, J. P. Jurado, J. S. Reparaz, J. Li, L. Pan, C. F. J. Faul, I. P. Parkin, G. Sankar, S. Matsuishi, M. Campoy-Quiles, D. O. Scanlon, M. A. Zwijnenburg, O. Fenwick and B. C. Schroeder, *Adv. Funct. Mater.*, 2020, **30**, 2003106.

19 K. Ueda, Y. Yamada, T. Terao, K. Manabe, T. Hirai, Y. Asaumi, S. Fujii, S. Kawano, M. Muraoka and M. Murata, *J. Mater. Chem. A Mater.*, 2020, **8**, 12319–12322.

20 Y. Wang, Q. Yao, S. Qu and L. Chen, *Journal Wuhan University of Technology, Materials Science Edition*, 2022, **37**, 760–764.

21 Y. Hu, G. Rivers, M. P. Weir, D. B. Amabilino, C. J. Tuck, R. D. Wildman, O. Makarovsky and S. Woodward, *Electronic Materials Letters*, 2024, **20**, 150–157.

22 R. Yura, T. Itoh, M. Ishihara, D. Suzuki, Y.-C. Kuo, S. Nagata, T. Yumura, S. Hosokawa, M. Murata and Y. Nonoguchi, *J. Mater. Chem. A Mater.*, 2025, **13**, 33685–33691.

23 N. Toshima and Y. Shiraishi, *Pure and Applied Chemistry*, 2020, **92**, 967–976.

24 N. Toshima, K. Oshima, H. Anno, T. Nishinaka, S. Ichikawa, A. Iwata and Y. Shiraishi, *Advanced Materials*, 2015, **27**, 2246–2251.

25 P. Sheng, Y. Sun, F. Jiao, C. Liu, W. Xu and D. Zhu, *Synth. Met.*, 2014, **188**, 111–115.

26 P. Sheng, Y. Sun, F. Jiao, C. Di, W. Xu and D. Zhu, *Synth. Met.*, 2014, **193**, 1–7.
